# Recommendations by the Spanish Society of Epidemiology and Oral Public Health (SESPO) for the healthcare adaptation of public health dental clinics in Spain during the COVID-19 pandemic

**DOI:** 10.4317/jced.57937

**Published:** 2020-12-01

**Authors:** María del Carmen Trullols-Casas, Verónica Ausina-Márquez, Yolanda Martínez-Beneyto, Elena Martínez-Sanz, Eulalia Llamas-Ortuño, Antonio Expósito-Delgado, Teresa Almerich-Torres, Victoria Mateos-Moreno, José-Maria Blanco-González

**Affiliations:** 1 DDS. Dental Public Health. Balearic Health Service, SESPO auditor board member; 2 DDS. PhD. Dental Public Health. Valencian Health Service. SESPO President; 3 DDS, PhD. Contracted Doctor Professor. Unit of Preventive & Community Dentistry. University of Murcia, Spain. SESPO Vice-President; 4 DDS. PhD. Contracted Doctor Professor. Unit of Anatomy. Faculty of Medecine. Complutense University of Madrid, Spain. SESPO Secretary; 5 DDS. Dental Public Health. Castilla-La Mancha Health Service, Spain. SESPO board member; 6 DDS. Dental Public Health. Andalusian Health Service, Spain. SESPO board member; 7 DDS, PhD. Associated Professor. Unit of Preventive & Community Dentistry. University of Valencia, Spain. SESPO board member; 8 DDS, PhD. Associated Professor. Unit of Preventive & Community Dentistry. Complutense University of Madrid, Spain. SESPO board member; 9 MD, DDS, PhD. Chair of Dental Public Health in Oviedo Region. Asturian Health Service, Spain. SESPO Treasurer

## Abstract

**Background:**

In March 2020, the World Health Organization (WHO) declared the COVID-19 pandemic and, a few days later, the Spanish Government declared a State of Emergency and the population lockdown. This crisis situation crisis forced deep changes in health care. At dental care level, it became necessary for both public health services and private consultations to plan changes to enable them to face this healthcare challenge.

**Material and Methods:**

SESPO and the General Council of Dentists of Spain (CGDE) appointed a Working Group to prepare a protocol for dental clinics after the lockdown stage. Continuing with this teamwork task, a series of recommendations addressed to public health managers and the dental workforce were agreed, according to the COVID-19 protection protocols, with the evidence available at the time of their preparation.

**Results:**

The SESPO Working Group prepared a schedule with recommendations to be taken. The CGDE presented this document to the Ministry of Health, Consumption and Social Welfare, and SESPO emailed it to all the Health Councils of the autonomous regions. The document was also uploaded to the CGDE and SESPO websites and was emailed to all SESPO associated members.

**Conclusions:**

Keeping in mind the existing territorial variation, both at the organization level of dental public health services, and at the care level (especially in child preventive programs and care for pregnant women), this health crisis has highlighted the importance of teamwork. It is necessary to unify the standards for all dental health care units in the national territory in challenging times.

** Key words:**COVID-19, Dental public health, dental care, dentistry, primary care, infection, SARS-CoV-2.

## Introduction

**Figure 1:**
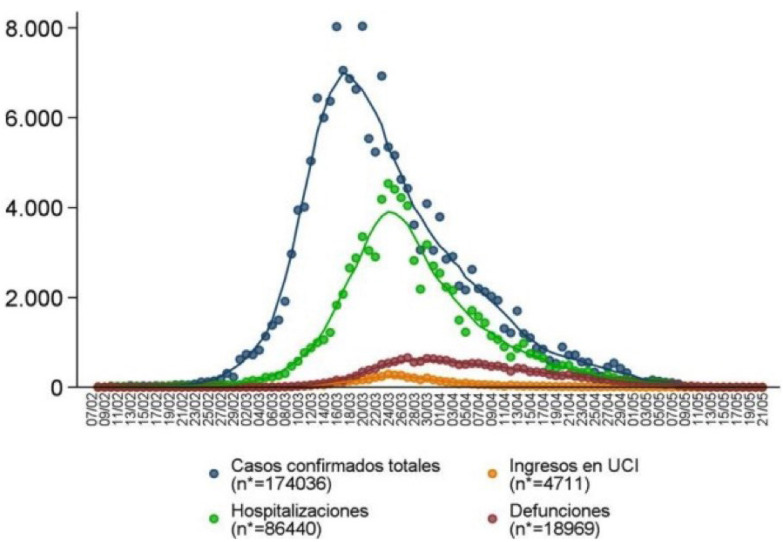
Epidemic curve of COVID-19 cases according to severity. COVID-19 cases notified to RENAVE (National Network for Epidemiological Surveillance). Taken from: COVID-19 in Spain on May 21, 2020. COVID-19 TEAM. RENAVE. CNE. CNM (ISCIII). * Original figure of the Ministry. Casos confirmado totales: Total confirmed cases Hospitalizaciones: Hospitalizations Ingesos en UCI: Admission to intensive care unit Defunciones: Deaths.

On March 11, 2020 the World Health Organization (WHO) declared the novel coronavirus COVID-19 outbreak a global pandemic. In Spain, the epidemic spread (Fig. 1) led the Central Government to declare a State of Emergency on March 14, with tough containment measures (1). Most citizens were homebound, banned from working and non-essential moving about. This stringent lockdown period meant that almost all dental care came to a standstill in the country. Face-to-face dental emergency care was only allowed when personal protection equipments (PPE) were available for dental health workers and appropriate safety measures for patients were put in place (2),. The telephone helpline for urgent dental assistance was introduced. In addition, in many Primary Care centres, the Bucco-Dental Health Units (BDHU) workers carried through supporting functions in the Primary Care Team (PCT).

Subsequently, the central Spanish Government planned successive transition phases to a “new normality” within the pandemic, following WHO factors for lifting restrictions outlined on April 16, and the EU Parliament resolution on April 17 (3). Each one of the nineteen autonomous regions would relax restrictions at a different pace, depending on the severity of its outbreak. The transition phases would allow the incorporation of new organizational and healthcare changes to those already introduced during the outbreak.

The protective protocol against COVID-19 forced dental teams to take costly and complicated safety measures, and to put them into practice in a short time. They meant: modifying the usual way that patients access the health centre and dental care units, changing the way of caring patients in the dental clinic and varying how some dental treatments were carried out. (4-6).

In proposing specific lines of action and offering useful references for the profession as a whole, the Spanish Society of Epidemiology and Oral Public Health (SESPO) and General Council of Dentists of Spain (CGDE) appointed a Working Group to prepare a protocol for dental clinics after the lockdown stage (7). It was also given the creation of an epidemiological observatory on the situation of dental workers for a year and following the pandemic. The first survey was carried out in Spain during the outbreak, in which 6,470 dental professionals (4,200 dentists, and 2,170 hygienists and nurse assistants) took part (8).

Continuing with this teamwork task, a series of recommendations addressed to public health managers and the dental workforce were agreed, with the intention of facilitating the best possible dental care according to the COVID-19 protection protocols, with the evidence available at the time of their preparation (9).

## Material and Methods

The SESPO Working Group prepared a schedule with recommendations to be taken (Table 1), which should be adapted depending on the severity of the outbreak in each autonomous region, and the healthcare demand in each Health Service, Health Area, and dental clinic. The CGDE presented this document to the Ministry of Health, Consumption and Social Welfare, and SESPO emailed it to all the Health Councils of the autonomous regions. The document was also uploaded to the CGDE and SESPO websites and was emailed to all SESPO associated members. Some public health managers appointed working groups with dental professionals to adapt these recommendations to the situation in their health area. In other cases, the dental workers organised working groups on their own to transmit and adapt these proposals to their local health managers due to the absence of a guidance coming from the public health directors for the reorganisation of BDHUs during the COVID-19 pandemic.

**Table 1:**
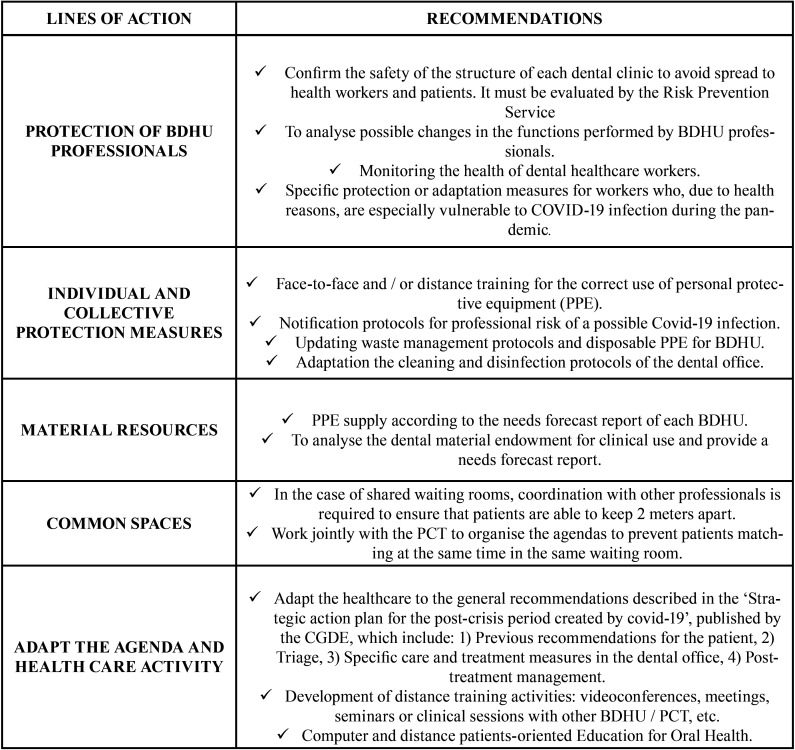
SESPO General Recommendations.

The patient’s access to dental care changed compared to that before the outbreak. Due to the social lockdown for theCOVID-19, it was established that it should be only by telephone appointment and scheduled face-to-face appointment. Thus, patients could be minimally treated without leaving their homes, and a prior triage could always be carried out to find out if either the patient suffered from COVID-19 or had recent symptoms or contact with COVID-19 patients in the last 14 days. Unscheduled face-to-face appointments in which no prior triage had been performed were minimized.

In all phases, referrals to hospital centres for specialized care (Maxillofacial Surgery, Pathological Anatomy, Radiodiagnosis, etc.) should be done by fax or e-mail. Each case should be carefully analyzed and unnecessary movements of patients should be avoided, especially in rural referral centres far from the BDHU and hospitals, scheduling a face-to face appointment only when it was considered necessary by the dental professional.

The recommendation to resolve the greatest number of dental care treatments at each appointment was reinforced to reduce population movement.

Three phases were defined:

a) Strict confinement phase: Urgent dental problems were attended by phone or with exceptional face-to-face appointments. Dental workforce were located either at home, going to the BDHU when an in-person emergency required it, or in the health centre. The face-to face dental care was limited to those emergency situations when non-care could clearly harm the patient. If the local COVID-19 outbreak progression and the local public health organization required it, the BDHU workforce could carry out support functions to the Primary Care Team within the health centre.

The community bucco-dental health preventive programs were temporarily suspended and their corresponding schedules were cancelled.

b) First transition phase for restarting the activity: BDHU workforce should be in the health centre. In case of a dental emergency, the patient should contact the Health Care Centre either by phone or, in some cases as urgent in-person appointment. The schedule for face-to-face programmed appointments should be controlled by the BDHU professionals. Patients who contacted the administrative public health centre staff, by phone or in-person, requesting an appointment, would be included in a list. The professionals of the BDHUs would phone each patient of this list for a triage and, in case it would be necessary, would schedule a face-to-face programmed appointment.

At this phase, Child Bucco-Dental Health Programs (CBDHP) and Pregnant Women Oral Care Programs (PWOHP) would not be restarted.

The dental care schedule in this phase could be organised into two different blocks: the telephone appointment block and the oral treatment (OT) face-to-face appointment block. The dentist would attend to the telephone block, which may or may not be urgent, previously evaluating the patient’s medical record, the Covid-19 triage, and the reason for a dental care request expressed by the patient. The evaluation of all these parameters would decide whether the patient is scheduled for the oral treatment (OT) schedule or not.

The OT appointment block should contain appointments of at least 20-30 minutes (OT-1), and at least 10 minutes for non-surgical care (OT-2).

c) Second transition phase or adaptation of the activity. Patients with emergencies should be cared in the same way described in the first transition phase. In this phase, CBDHP and PWOHP preventive care patients should be scheduled. Those patients that either had treatments started before strict confinement, or those that were considered as high risk for oral pathology, should be seen.

The treatment schedule would be structured in three different blocks: The telephone appointment block, the OT appointment block, and the CBDHP block with expandable appointments every 15 minutes. In the OT block the patients of the PWOHP would also be seen if the dentist considered it necessary in the previous telephone triage.

Depending on the local characteristics of each BDHU, some days could be organised exclusively dedicated to either OT block or CBDHP blocks, instead of dividing the workday between them.

**Figure 2:**
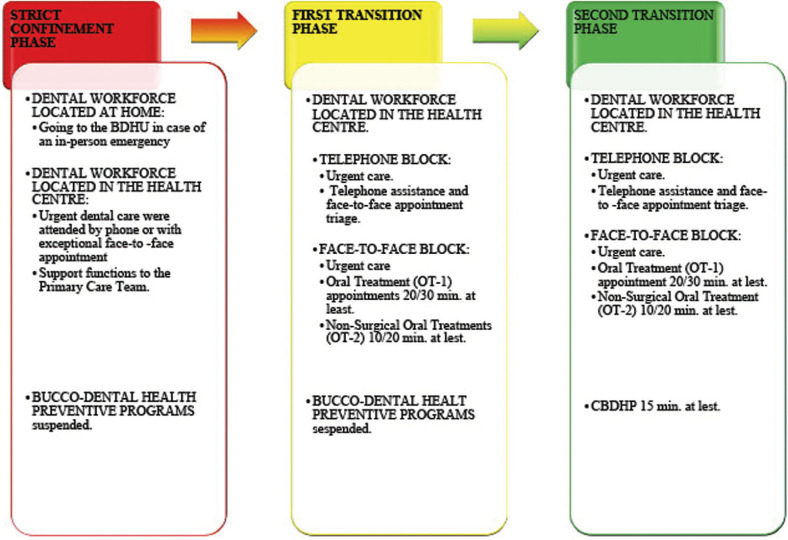
Generic recommendations for dental care in public health centres.

The following action schedule is included as a generic proposal (Fig. 2), which should be adapted according to the changing COVID-19 epidemiological situation and the healthcare pressure registered by each public health region.

## Results

In several public health regions, the collaboration between the healthcare directors and the BDHU workforce to assess the recommendations presented by SESPO-CGDE has allowed the improvement of the dental healthcare organization.

The management of this pandemic has introduced new concepts that have turned out to be very useful in Primary Care. The most significant was the telephone dental healthcare, which was essential during the strict confinement phase to minimize patient movements. In the transition phases, this kind of patient care was maintained, because it not only allowed a triage, but also permitted many possible positive applications, despite what seemed impersonal care. The telephone consultations were greatly promoted, which could be used for both health education, pharmacological prescriptions, monitoring the evolution of a pathology, preoperative instructions, etc. It has been effective, most patients have learned to use it, and it has reduced face-to-face care appointments.

## Discussion

-Challenges addressed

Keeping in mind the existing territorial variation, both at the organization level of dental public health services, and at the care level (especially in child preventive programs and care for pregnant women), this health crisis has highlighted the importance of teamwork. Both from the PCT level to the CGDE and SESPO collaboration level, which have met the need to unify the standards for all BDHUs in the national territory in challenging times.

The collaborative working allowed everyone to reach consensus about the recommendations and let them be reported as quickly as the crisis situation required, reaching to the maximum organisations of sanitary government and public health management services, as well as to dental professionals nationwide.

-Future implications

The COVID-19 pandemic declaration forced all health care workers to continually update knowledge in order to adapt their routine healthcare to new evidence that is emerging. SESPO and the CGDE maintain the workgroup to provide updates on the recommendations once they are agreed.

It is of vital importance for society to learn the new way of accessing Primary Care health services. Very often before the crisis, urgent face-to-face appointments weren’t really for a true emergency. From the beginning of the health crisis, insistent messages have been launched to the population through the media telling them that before going to the health centres, you should make contact by phone. It is necessary to maintain education for the proper use of health resources to generate positive attitude changes in several generations.

New technologies have been an important point to transmit and receive information in a short time, and also to apply in healthcare practice. In some autonomous regions, healthcare workers located at home were licensed to get safe access to the healthcare computer network to be able to provide telephone assistance from home. This would reinforce the idea that telemedicine can also be applied in dentistry.

It would be interesting to promote the Minimum Intervention Dentistry in public dental care, with training and the incorporation of materials that simplify dental techniques, especially when it is important to optimize time in healthcare and to minimize aerosol generating treatments (e.g. Silver Diamine Fluoride, Hydrophilic Sealants, or Glass Ionomers).

The cornerstone to manage public dental care during COVID-19 pandemic is to be prepared to modify care standards, assuming the possibility of revamping the BDHUs teams during the transition phases, as an increase in the care pressure is expected in the future. This may be due to various factors: many patients haven’t received face-to-face care for a period of time, non-urgent treatments were postponed, and the great economic crisis that unfortunately accompanies the health crisis, which makes it difficult for patients to afford their dental conservative treatments in private dental clinics.

The channels of communication and information between public health services managers and the dental care workforce must be maintained and improved in order to continue evaluating, proposing, and integrating dynamic solutions in situations of health crises.

This improvement in the collaboration between healthcare directors and front-line professionals would enable the standardization of all dental activity carried out in the different health services in Spain and implement processes of continuous improvement of the healthcare and training aspects of BDHU professionals, adding them to the quality policies of all public health services.

## Conclusions

This situation may have taught us that when dental care programs are weakened, it is important to organise, maintain and investigate ways of collaboration to guarantee dental care to the population.

The health response to current health challenges must improve in the future with an European global approach to surveillance, monitoring, information, research and the proposal of joint solutions. Oral health should not be an exception in this integration.
